# Understanding the Associations Between Adverse Childhood Experiences and Spiritual Well‐Being Among Turkish University Students: Testing the Mediating Roles of Rumination and Forgiveness

**DOI:** 10.1002/brb3.70923

**Published:** 2025-10-20

**Authors:** Gülçin Güler Öztekin, Hurizat Hande Turp, Najmah Abdallah Alzahrani, Juan Gómez‐Salgado, Murat Yıldırım

**Affiliations:** ^1^ Department of Psychology, Faculty of Science and Letters Agri Ibrahim Cecen University Agri Turkey; ^2^ Department of Guidance and Psychological Counseling, Faculty of Education Agri Ibrahim Cecen University Agri Turkey; ^3^ Department of Psychology Taif University Taif Saudi Arabia; ^4^ Department of Sociology, Social Work and Public Health, Faculty of Labour Sciences University of Huelva Huelva Spain; ^5^ Safety and Health Postgraduate Program Universidad Espíritu Santo Guayaquil Ecuador; ^6^ Psychology Research Center Khazar University Baku Azerbaijan

**Keywords:** adverse childhood experiences, forgiveness of others, rumination, self‐forgiveness, spiritual well‐being

## Abstract

**Purpose:**

The formation of spiritual well‐being is essential for individuals. The purpose of this study was to investigate the mediating roles of rumination and forgiveness in the link between adverse childhood experiences and spiritual well‐being in both independent and sequential paths.

**Method:**

The sample of this study consisted of 1138 university students (71.4% females; *M*  =  22.06, SD  =  2.43). The results showed that adverse childhood experiences were negatively associated with spiritual well‐being. Rumination mediated the link between adverse childhood experiences and spiritual well‐being. Self‐forgiveness and forgiveness of others acted as mediators in this relationship. Rumination—self‐forgiveness and rumination—forgiveness of others sequentially mediated the relationship.

**Finding:**

These results highlight that adversities experienced during childhood are associated with higher levels of rumination, and these repetitive thoughts inhibit forgiveness, leading to reduced spiritual well‐being.

**Conclusion:**

This study suggests that minimizing rumination and promoting forgiveness may be useful strategies to enhance spiritual well‐being for university students who have experienced adverse life events.

## Graphical abstract

This model illustrates the associations between adverse childhood experiences (ACEs) and spiritual well‐being among Turkish university students, highlighting the mediating roles of rumination, self‐forgiveness, and forgiveness of others. ACEs were directly associated with lower spiritual well‐being. Rumination mediated this relationship independently, and in sequential pathways through reduced self‐forgiveness and forgiveness of others. These findings suggest that interventions targeting rumination and promoting forgiveness may buffer the negative impact of ACEs on spiritual well‐being.

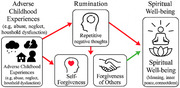

## 1 Introduction

The state of being that encompasses positive emotions, cognitions, and behaviors in one's relationships with self, community, nature, and the transcendent constitutes spiritual well‐being. In Fisher's multidimensional model, spiritual well‐being is concerned with the degree of harmony people have in their relationships with oneself (personal), with others (communal), with nature (environmental), and with God (or a transcendental other) (Fisher 2010). The personal domain encompasses how a person relates to oneself in terms of meaning, purpose, and values in life. The communal domain includes the quality and depth of interpersonal relationships between self and others and is concerned with love, hope, justice, and faith in humanity. The environmental domain includes care and nurturing for the physical and biological world through a sense of wonder, awe, and unity with the environment. The transcendental domain encompasses the self's relationship to something or someone beyond the human level, such as God, transcendent reality, or a cosmic force, and involves belief in, adoration, and worship of the mysterious source of the universe (Gomez and Fisher 2003). Considering the perception of spirituality in Turkish culture, it has been determined that spirituality covers these four areas and that religious activities such as relationship with God, praying, and reading the holy book, the Quran, significantly affect the areas of spiritual well‐being (Fisher and Coskun 2013). In addition, spiritual well‐being nurtures and celebrates wholeness, and provides the individual with love, identity, inner peace, contentment, respect, and a sense of purpose and direction in life (Gomez and Fisher 2003). This supports psychological and subjective well‐being (Chirico et al. 2023; Makkaoui et al. 2024; Özdemir et al. 2023; Unterrainer et al. 2010). In cases where physical and mental health are at risk, it is very important to ensure spiritual well‐being during treatment (Cheng et al. 2019; Shaygan and Shayegan 2019). While life satisfaction, quality of life, hope, social support, mental health, psychological well‐being, and spiritual coping are factors that contribute to spiritual well‐being (Rahmat et al. 2022) and positive mental health (Şanli et al. 2024), adverse childhood experiences are a factor that hinders the formation of spirituality (Santoro et al. 2016).

Adverse childhood experiences are childhood events that occur in a child's family or social environment, are of varying degrees of severity, are often chronic, and cause harm or distress. Adverse experiences encompass experiences of harm to the child through substance abuse, abuse or neglect, criminal activity, exposure to domestic violence, or family dysfunction. These experiences impair the child's physical or psychological health and development (Kalmakis and Chandler 2014). More importantly, multiple adverse childhood experiences pose risks for future generations, such as violence, mental illness, and substance abuse (Hughes et al. 2017). In addition, excessive negative experiences harm the well‐being of individuals (Mosley‐Johnson et al. 2019). As the number of these experiences increases, the protective role of spiritual strength decreases and may even reverse, suppressing the development of spiritual well‐being (Shin et al. 2024). These negative experiences also disrupt the spirituality that is formed by understanding oneself, finding meaning in one's life, and achieving inner peace, and lead to psychological problems (Freeny et al. 2021). On the other hand, positive and pleasant experiences in the early years of life enhance spiritual well‐being (Maral et al. 2024). These studies suggest that children should be exposed to positive experiences instead of negative ones during childhood for the development of spiritual well‐being. Based on the detrimental effect of adverse childhood experiences on spiritual well‐being, we proposed our first hypothesis as follows:

H1: Adverse childhood experiences have a direct effect on spiritual well‐being.

### 1.1 Rumination as a Mediator

Rumination is a cognitive process that refers to various types of repetitive, event‐related thinking, including inference, problem solving, recall, and anticipation (Martin and Tesser 2013). While early adversities and environmental factors increase the possibility of rumination, rumination increases the occurrence of psychological problems, prevents problem solving, impairs concentration, causes impulsive behaviors and physical illnesses (Watkins and Roberts 2020). Rumination may be a way of responding to the consequences of adverse childhood experiences, such as depression. Individuals who ruminate dwell on problems and their feelings about those problems without taking action, rather than actively problem‐solving to change the circumstances surrounding the symptoms (Nolen‐Hoeksema et al. 2008). Traumatic events and their consequences may lead to rumination that interferes with a survivor's hopeful mindset, and these ruminative thoughts may inhibit individuals from achieving their goals and the problem‐solving (Muñoz and Hanks 2021). Thus, adverse childhood experiences may increase rumination. In addition, in difficult situations, the repetitive form of negative thinking may prevent the divine values that individuals attribute spiritual importance to from being in a consistent and balanced integration with society and themselves (Taşdemir et al. 2025). Therefore, rumination may reduce spiritual well‐being. Previous literature has also provided evidence for the mediating role of rumination. For example, minimizing rumination was identified as a useful strategy to support mental health in adolescents experiencing negative life events (Boyes et al. 2016). Low social support increased ruminative thinking, which in turn decreased spiritual well‐being (Li et al. 2022). These studies have shown the negative contribution of rumination in the impact of adverse life events in childhood on spiritual well‐being, and we proposed our second hypothesis as follows:

H2: Rumination has a mediating role in the link between adverse childhood experiences and spiritual well‐being.

### 1.2 Forgiveness as a Mediator

As social beings, people interact at every stage of life, making it inevitable for some problems to occur in interpersonal relationships. Forgiveness, which is one of the reactions to these hurt or traumatic situations experienced in interpersonal relationships, plays an important role in repairing relationships and providing benefits to the individual (Aziz and Yıldırım 2020; Strelan et al. 2013). Self‐forgiveness refers to releasing anger at oneself for a perceived offence or wrongdoing and increasing compassion and love toward the self (Enright 1996), while forgiveness of others involves the deliberate decision to let go of anger and resentment toward a transgressor (Worthington 2019). Forgiving others allows people to confront their anger voluntarily and look at the transgressor from a more compassionate perspective (Enright and Fitzgibbons 2015). People who forgive themselves and others have lower stress levels, better perceived health, and greater life satisfaction (Toussaint et al. 2023). Individuals who have had adverse childhood experiences may have difficulty forgiving themselves or others due to the negative consequences of these experiences, such as anger, negative emotions, and blame (Taylor 2020). Both self‐forgiveness and forgiveness of others may act as coping mechanisms that buffer the impacts of parents' adverse childhood experiences on children's same experiences (Skolnick et al. 2023). Forgiveness can also promote post‐traumatic personal growth for adverse childhood experience survivors (Amaranggani and Dewi 2022), which includes enhancing spiritual well‐being (Feng et al. 2024). Forgiveness supports the individual's spiritual integrity and well‐being by providing inner peace, assisting in the search for meaning in life, reducing negative emotions, and improving the individual's relationship with the self, other people, and the divine (Webb et al. 2012). Furthermore, adverse childhood experiences reduced forgiveness, which increased the possibility of mental health problems, providing evidence for the mediating role of forgiveness (Rahmandani et al. 2022). Forgiveness increased well‐being by reducing the long‐term effects of childhood bullying among individuals who developed spirituality (Theodora et al. 2023). Negative experiences in childhood can cause individuals to feel anger toward themselves or toward those who caused these experiences. Self‐forgiveness helps individuals release their anger toward themselves, while forgiving others helps them confront the people who caused the negative experiences and look at the offender with a more compassionate perspective (Enright and Fitzgibbons 2015; Enright 1996). When this negativity is eliminated, the individual can achieve inner peace, accelerate the building of relationships with themselves, their environment, and God, and achieve meaning in life; in other words, their spiritual well‐being can increase. Therefore, these two distinct processes may mediate the association between adverse childhood experiences and spiritual well‐being. Since the findings of the above‐mentioned studies suggest that forgiveness is a means to improve coping, we proposed our third hypothesis as follows:

H3: Self‐forgiveness and forgiveness of others have mediating roles in the link between adverse childhood experiences and spiritual well‐being.

### 1.3 Rumination and Forgiveness as Chain Mediators

Transactional model of stress and coping, a cognitive‐relational theory of emotion and coping, posits that the individual and the environment are constantly interacting. Humans constantly evaluate what is happening to them from the standpoint of its significance for their well‐being, and the model focuses on how the person evaluates these events (cognitive processes). When people encounter a stressor, they evaluate whether the situation is dangerous or harmful in the primary appraisal process. In the secondary appraisal process, they think of how they can cope with the situation by applyingtheir existing resources, such as coping skills, support system, and information. They can reduce stress and adapt to the situation with problem‐focused or emotion‐focused coping strategies (Lazarus and Folkman 1987). Individuals with adverse childhood experiences may attempt to adapt to the situation with avoidant emotion‐focused coping, which involves strategies that serve to reduce one's negative emotional state in response to a stressor but do little to resolve the actual stressor (Sheffler et al. 2019). Unresolved stressors resulting from these experiences may lead to repetitive negative thinking, such as rumination (Mansueto et al. 2021). As individuals' repetitive thoughts increase, it becomes more difficult for them to forgive themselves and those who have hurt them (Karremans and Smith 2010; Onal and Yalcın 2017). Reducing rumination is a process that facilitates forgiveness (Wu et al. 2019). Self‐forgiveness and forgiveness of others can be considered as an emotion‐focused coping approach that can be addressed within the scope of the transactional theory of stress and coping (Toussaint et al. 2017). Furthermore, forgiveness is important throughout life, from childhood to old age, and the process of forgiveness should be nurtured for their spiritual development (Flanagan et al. 2012). This theory and previous studies suggest that reducing rumination and cultivating forgiveness may help enhance spiritual well‐being among individuals experiencing childhood adversities. Therefore, we proposed our fourth and fifth hypotheses as follows:

H4: Rumination and self‐forgiveness have a chain mediating role in the link between adverse childhood experiences and spiritual well‐being.

H5: Rumination and forgiveness of others have a chain mediating role in the link between adverse childhood experiences and spiritual well‐being.

This study presents a unique model examining the cognitive‐emotional mechanisms that explain the impact of adverse childhood experiences on spiritual well‐being in a Turkish cultural context. While the literature contains numerous studies on the psychological and emotional consequences of adverse childhood experiences, research exploring the implications of these effects on spiritual well‐being is limited. Specifically, no studies have been found that examine internal processes such as rumination and forgiveness of self and others within a serial mediation model. In addition, spiritual well‐being can be considered as an outcome and a process variable, and researchers have highlighted the need for further research to identify factors that contribute to or undermine spiritual well‐being (Bredle et al. 2011; Chen et al. 2017). The current research specifically explores how forgiveness tendencies relate to rumination to understand how adverse childhood experiences shape an individual's spiritual orientation and meaning in life. This research thus reveals concrete cognitive‐emotional pathways that can be considered in psychological intervention programs that support spiritual well‐being. Furthermore, testing this model in a Turkish sample provides a cultural perspective by examining the role of rumination and forgiveness on spiritual processes. In conclusion, this study contributes theoretically and practically to the literature by comprehensively considering how adverse early life experiences can impact spiritual well‐being through an individual's internal resources and cognitive processes.

## 2 Method

### 2.1 Participants

1138 Turkish university students participated in this study. A total of 813 students were female (71.4%), and 325 students were male (28.6%). The students’ ages ranged from 18 to 35, with a mean age of 22.06 years (SD =  2.43). Most of the participants were juniors (*n* = 307), followed by seniors (*n* = 304), sophomores (*n* = 267), freshmen (*n* = 178), graduate students (*n* = 41), preparatory students (*n* = 23), and fifth‐year students (*n* = 18). 138 students perceived their childhood as very good, 654 students perceived it as good, 207 students perceived it as neutral, 107 students perceived it as bad, and 32 students perceived it as very bad. These results are presented in Table 1.

**TABLE 1:** Demographic characteristics of the participants.

Participants	*N*	%
**Gender**		
Female	813	71.4
Male	325	28.6
**Education**		
Preparatory students	23	2.0
Freshman	178	15.6
Sophomore	267	23.5
Junior	307	27.0
Senior	304	26.7
Fifth‐year students	18	1.6
Graduate students	41	3.6
**Evaluation of childhood experiences**		
Very good	138	12.1
Good	654	57.5
Neutral	207	18.2
Bad	107	9.4
Very bad	32	2.8
**Total**	1138	100.0

### 2.2 Measures

#### 2.2.1 Adverse Childhood Experiences Scale

The scale was developed after researchers from the Centers for Disease Control and Prevention and Kaiser Permanente worked to develop and study the “Adverse Childhood Experiences” scale to measure adverse early life experiences (Felitti et al. 1998). Gündüz et al. (2018) adapted the scale to Turkish culture to determine the presence of adverse experiences such as emotional, physical, and sexual violence, abuse, and emotional and physical neglect in the first 18 years of an individual's life. The scale is unidimensional and includes ten items with a yes–no response. The sample item is “Did a parent or other adult in the household …Often or very often swear at, insult, or put you down? Often or very often act in a way that made you afraid that you would be physically hurt?” The score that can be obtained from the scale varies between 0 and 10. As the score increases, exposure to adverse childhood experiences also increases. The Cronbach's alpha value was calculated as 0.74 (Gündüz et al. 2018). In this study, the Cronbach's alpha value was 0.71.

#### 2.2.2 Ruminative Response Scale

The scale was developed by Treynor et al. (2003), shortened by Nolen‐Hoeksema and Morrow (1991), and adapted to Turkish culture by Erdur Baker and Bugay (2012). The scale consists of two subscales, brooding and reflection, and includes ten items scored on a 4‐point Likert‐type scale ranging from 1 (never) to 4 (always). The sample item is “How often do you think”, “Why do I have problems and other people don't?” As the scores increase, this indicates that individuals engage in ruminative thinking more. The Cronbach's Alpha value was calculated as 0.85 (Erdur Baker and Bugay 2012). In this study, the Cronbach's Alpha value was 0.84.

#### 2.2.3 State Self‐Forgiveness Scale

The scale was developed by Wohl et al. (2008) and adapted to Turkish culture by Havare and Gizir (2020). The scale consists of three subscales: emotions and behaviors, positive beliefs, and negative beliefs, and includes 12 items scored on a 4‐point Likert‐type scale ranging from 1 (never) to 4 (always). The sample item is “When I think that something I did was wrong, I believe that I am a bad person.” As the scores increase, the level of self‐forgiveness of individuals also increases. The Cronbach's Alpha value was calculated as 0.87 (Havare and Gizir 2020). In this study, the Cronbach's alpha value was 0.86.

#### 2.2.4 RYE Forgiveness Scale

The scale was developed by Rye et al. (2001) and adapted to Turkish culture by Havare and Gizir (2020). The scale consists of three subscales: negative thoughts and behaviors, positive thoughts and behaviors, and emotions, and includes 15 items scored on a 5‐point Likert‐type scale ranging from 1 (strongly disagree) to 5 (strongly agree). The sample item is “I wish for good things to happen to the person who wronged me.” As the scores increase, the level of forgiveness of individuals toward others also increases. The Cronbach's Alpha value was calculated as 0.75 (Havare and Gizir 2020). In this study, the Cronbach's alpha value was 0.77.

#### 2.2.5 Spiritual Well‐Being Scale

The scale was developed by Bredle et al. (2011) and adapted to Turkish culture by Arslan and Yıldırım (2021). The scale is unidimensional and includes five items on a 5‐point Likert‐type scale ranging from 0 (not at all) to 4 (very much). The sample item is “I feel a sense of purpose in my life.” As the scores increase, the level of spiritual well‐being also increases. The Cronbach's Alpha value was calculated as 0.85 (Arslan and Yıldırım 2021). In this study, Cronbach's alpha value was 0.70.

### 2.3 Procedure

The Ethics Committee of (blinded for review) University approved this study (Ethic Code: 124705). The individuals were recruited via convenient sampling. The link for this study was prepared through Google Forms. Participants were informed about the study aims, procedures, and their rights before and after participation in the survey preface. Only volunteers were allowed to proceed to answer. Consent was obtained from each participant before completing the survey.

### 2.4 Data Analyses

Before testing the mediation model, preliminary analyses such as descriptive statistics and correlation analysis were conducted. Kurtosis and skewness scores were evaluated to examine normality assumptions (Tabachnick et al. 2013). Pearson correlation analysis was conducted to investigate relationships between the study variables. To test the proposed mediation model, we used PROCESS‐Macro v4.2 with model 81 and evaluated the results with standardized and unstandardized regression coefficients and squared multiple correlation values. We evaluated the indirect effects of mediators at 95% confidence intervals using a bootstrapping technique with 5.000 bootstrap samples. All analyses were run using SPSS version 27.

### 2.5 Common Method Bias

The current study involved self‐report measures that may be subject to response bias and social desirability effects, which could lead to common method bias. This could negatively impact the validity of the study. To eliminate bias, exploratory factor analysis was conducted using Harman's single‐factor test. This test involves clustering all variables in the study under a single factor. A single factor explaining 50% or more of the total variance shows a homogeneous structure (Harman 1976). In this study, the variance explained was 14.65%, indicating that the study did not suffer from common method bias.

## 3 Results

Preliminary analysis indicated the normal distribution with skewness and kurtosis values (see Table 2). The findings of the correlation analysis showed that adverse childhood experiences had a significant positive correlation with rumination, while significant negative correlations with self‐forgiveness, forgiveness of others, and spiritual well‐being. Rumination had significant negative correlations with self‐forgiveness, forgiveness of others, and spiritual well‐being. Self‐forgiveness and forgiveness of others had a significant positive correlation with spiritual well‐being. These results are presented in Table 2.

**TABLE 2:** Descriptive statistics, skewness, kurtosis, and correlations.

Variables	M	SD	Skewness	Kurtosis	Correlation				
					1	2	3	4	5
1. Adverse childhood experiences	1.24	1.70	1.41	1.18	—				
2. Rumination	22.53	5.24	0.44	0.56	0.28**	—			
3. Self‐forgiveness	31.92	6.58	0.01	0.24	−0.15**	−0.16**	—		
4. Forgiveness of others	45.08	8.05	−0.14	0.84	−0.18**	−0.31**	0.27**	—	
5. Spiritual well‐being	12.19	3.63	−0.38	0.33	−0.26**	−0.37**	0.23**	0.29**	—

*Note*: *M* = mean, SD = standard deviations.

**p < 0.001.

**FIGURE 1:**
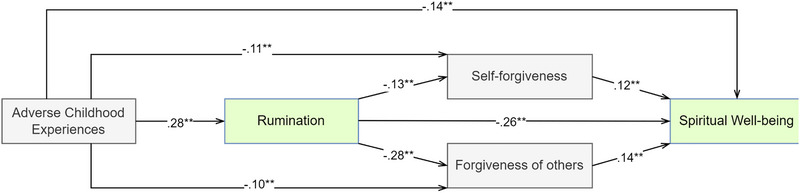
Standardized effects of the mediation model.

The findings of mediation analysis revealed that adverse childhood experiences were significantly associated with rumination (*β* = 0.86, *p* < 0.001), self‐forgiveness (*β* = −0.45, *i* < 0.001), and forgiveness of others (*β* = −0.49, *p* < 0.001). Rumination was significantly associated with self‐forgiveness (*β* = −0.17, *p* < 0.001) and forgiveness of others (*β* = −0.43, *p* < 0.001). Adverse childhood experiences (*β* = −0.30, *p* < 0.001), rumination (*β* = −0.18, *p* < 0.001), self‐forgiveness (*β* = 0.06, *p* < 0.001), and forgiveness of others (*β* = 0.06, *p* < 0.001) significantly predicted spiritual well‐being. They explained 20% of the variance in spiritual well‐being (see Table 3). The standardized effects for the mediation model are presented in Figure 1.

**TABLE 3:** Unstandardized coefficients for the mediation model.

Predictor	Outcome	Coeff.	SE	*t*	*p*	
Adverse childhood experiences	Rumination	0.86	0.08	9.89	0.00	*R* ^2^ = 0.07 *F* = 97.98; *p* < 0.001
Adverse childhood experiences	Self‐forgiveness	−0.45	0.11	−3.91	0.00	*R* ^2^ = .04 *F* = 24.59; *p* < 0.001
Rumination	Self‐forgiveness	−0.17	0.03	−4.48	0.00	
Adverse childhood experiences	Forgiveness of others	−0.49	0.13	−3.57	0.00	*R* ^2^ = 0.10 *F* = 68.51; *p* < 0.001
Rumination	Forgiveness of others	−0.43	0.04	−9.68	0.00	
Adverse childhood experiences	Spiritual well‐being	−0.30	0.05	−5.07	0.00	*R* ^2^ = 0.20 *F* = 74.65; *p* < 0.001
Rumination	Spiritual well‐being	−0.18	0.01	−9.25	0.00	
Self‐forgiveness	Spiritual well‐being	0.06	0.01	4.53	0.00	
Forgiveness of others	Spiritual well‐being	0.06	0.01	5.12	0.00	

*Note*: Coeff.  =  unstandardized coefficient; SE  =  standard error.

The direct effect of adverse childhood experiences on spiritual well‐being was −0.30 [−0.41, −0.18]. The indirect effect of adverse childhood experiences on spiritual well‐being through rumination (effect  =  −0.16, [−0.21, −0.11]), self‐forgiveness (effect  =  −0.03, [−0.05, −0.01]), and forgiveness of others (effect  =  −0.03, [−0.05, −0.01]) was found to be significant. The results of the serial mediation analysis showed that the indirect effect of adverse childhood experiences on spiritual well‐being through both rumination and self‐forgiveness (effect  =  −0.01, [−0.02, −0.01]), and rumination and forgiveness of others (effect  =  −0.02, [−0.03, −0.01]) was also significant (see Table 4).

**TABLE 4:** Total, direct, and indirect effects.

									95%	
Model pathway		Mediator				Outcome	Effect	BootSE	LLCI	ULCI
Adverse childhood experiences	→	Rumination	→			Spiritual well‐being	−0.16	0.02	−0.21	−0.11
Adverse childhood experiences	→	Self‐forgiveness	→			Spiritual well‐being	−0.03	0.01	−0.05	−0.01
Adverse childhood experiences	→	Forgiveness of others	→			Spiritual well‐being	−0.03	0.01	−0.05	−0.01
Adverse childhood experiences	→	Rumination	→	Self‐forgiveness	→	Spiritual well‐being	−0.01	0.01	−0.02	−0.01
Adverse childhood experiences	→	Rumination	→	Forgiveness of others	→	Spiritual well‐being	−0.02	0.01	−0.03	−0.01
Total indirect effect							−0.25	0.03	−0.32	−0.20
Direct effect							−0.30	0.05	−0.41	−0.18
Total effect							−0.56	0.06	−0.68	−0.44

*Note*: Number of bootstrap samples for percentile bootstrap confidence intervals: 5.000.

## 4 Discussion

Experiences of harm inflicted on a child through substance abuse, abuse or neglect, criminal activity, exposure to domestic violence, or family dysfunction can have long‐lasting effects on individuals and undermine their spiritual well‐being. Therefore, it is necessary to identify factors that can mitigate the impact of this adversity. The findings of this study indicated that adverse childhood experiences had a direct effect on spiritual well‐being. Participants who reported high negativity in childhood had low spiritual well‐being. Consistent with our results, emotional, physical, and sexual abuse and emotional and physical neglect in early life years increased the risk of spiritual struggles (Janů et al. 2022). Psychological maltreatment in childhood increased the levels of intolerance of uncertainty, impaired emotion regulation skills, and thereby reduced spiritual well‐being (Yilmaz and Satici 2024). Individuals’ history with their family had a significant impact on their subsequent spiritual experiences (Arslan 2025). These results emphasize the importance of spirituality and the damage that negative experiences can have on spirituality.

This study revealed that rumination had a mediating role in the link between adverse childhood experiences and spiritual well‐being. Individuals who encountered negative life events before the age of 18 reported that they engaged in more ruminative thoughts, which decreased their spiritual well‐being. Similarly, ruminative thought was identified as a mediator in the association between childhood maltreatment and trait mindfulness (Fox et al. 2024). Individuals who lacked social support during difficult times resorted to rumination more often, which in turn reduced spiritual well‐being (Li et al. 2022). These results show that rumination is a potential barrier to spiritual well‐being.

The results of this study showed that self‐forgiveness and forgiveness of others had mediating roles in the link between adverse childhood experiences and spiritual well‐being. Negative life events made it harder for individuals to forgive themselves and others, which lowered their spiritual well‐being. Previous research has demonstrated that forgiveness may help mitigate the adverse childhood experiences’ negative impact on well‐being. For instance, the link between victims’ appraisal of the offence and levels of well‐being was mediated by forgiveness, suggesting that individuals who forgive others tend to experience greater well‐being (Gismero‐González et al. 2020). Furthermore, after a transgression, apologizing or showing remorse has been determined to encourage forgiveness from both the victims and those around them (Yucel and Vaish 2021). However, it is important to note that forgiveness is influenced by how much the victim values his or her relationship with the perpetrator and the event. Membership in the same ethnocultural group, genetic relatedness, and history of productive interaction determine this value, while the enormity of the harm and the harmdoer, and benevolent approaches such as apologies, influence the emergence of forgiveness (McCauley et al. 2022). Turkish culture is largely nourished by the Islamic faith. Spirituality is related to values such as forgiveness, compassion, and tolerance, which support individuals in establishing more understanding, forgiving, and healthy relationships with one another. Spirituality in Turkish culture is formed through various traditional, religious, and cultural activities such as reading the Quran, embracing the morality of the Prophet Muhammad, trusting in God, praying, giving thanks, forgiving, and being patient, which strengthen individuals' relationship with their inner world, society, and God (Gürsu 2022). In Turkish culture, forgiveness is an essential principle for both individual virtue and social peace (Yılmaz and Tosun 2024). Therefore, forgiveness plays a key role in resolving conflicts within society and in personal development and supports spirituality.

This study also determined that rumination and self‐forgiveness, and rumination and forgiveness of others had chain mediating roles in the link between adverse childhood experiences and spiritual well‐being. Individuals who experienced adverse life events reported experiencing higher levels of repetitive thoughts, making it harder for them to forgive themselves and others, and this process decreased their spiritual well‐being. Similar to our results, anxious and avoidant attachment shaped by childhood experiences negatively affected forgiveness and subsequently marital satisfaction through excessive rumination and lack of empathy, providing evidence for the chain mediating roles of rumination and forgiveness (Chung 2014). Negativities experienced in childhood can profoundly affect biological, psychological, social, and spiritual health (Cooper and Wolfer 2024). Ruminating about these events that harmed the individual hinders the individual's ability to forgive. Additionally, self‐forgiveness and meaning work together to help people resolve their repetitive thoughts, learn more about themselves, make amends, and move toward healing their pain and the pain they have caused in others (Graham et al. 2017). From these results, we can infer that individuals who have had negative childhood experiences can gain different perspectives instead of repetitive thoughts, which leads to forgiveness and then spiritual well‐being.

### 4.1 Theoretical and Practical Implications

This study presents theoretical and practical implications. This study found that childhood adversities increase rumination, which negatively impacts forgiveness, thus reducing spiritual well‐being. These results align with the transactional model of stress and coping. This Lazarus's model is the prevailing conceptual framework for understanding the relationships among stress, coping, and their impact on individual health and well‐being. The impact of a particular stressor depends on the individual's appraisal of that stressor and the availability of coping responses (Lazarus and Folkman 1987). Adverse childhood experiences have cumulative and detrimental effects on health and well‐being across the lifespan, and these become stressors that elicit negative emotions, repetitive thinking, and situations (Mansueto et al. 2021). Alleviating anger and reducing rumination are important and intervening processes for developing forgiveness (Wu et al. 2019). Forgiveness can be a mediator and function as a coping mechanism that impacts health and well‐being (Toussaint et al. 2017). Spirituality was essential for enhancing well‐being through forgiveness (Gaventa 2013; Skalski‐Bednarz and Toussaint 2024). Therefore, therapeutic interventions can be developed to increase individuals’ awareness of repetitive thoughts and to encourage the process of forgiveness. In particular, mindfulness, cognitive behavioral therapy targeting cognitive restructuring, acceptance and commitment therapy aiming to accept challenging thoughts and emotions without suppressing them, empathy, and emotion regulation activities should be implemented in these intervention programs. Caregivers should be informed about the importance of early childhood experiences, the long‐term effects of childhood trauma, and the importance of spirituality.

### 4.2 Limitations

When evaluating the findings of this study, some limitations should be taken into consideration. This is a cross‐sectional study and does not allow any causal inference. Future studies should focus on longitudinal and experimental studies. The majority of participants were female, limiting the generalizability of the results. Researchers should repeat the study with a more balanced gender distribution.

## 5 Conclusion

In conclusion, since spiritual well‐being is a component of overall well‐being, promoting it is a priority. This study determined the inverse relationship between adverse childhood experiences and spiritual well‐being. Traumatic events can undermine an individual's perspective of self and the world, making it difficult to construct meaning, connect with a transcendent being, and seek inner peace. Rumination and forgiveness were mediators in this relationship. Adverse childhood experiences were associated with greater rumination, which prevented individuals from forgiving themselves and others, resulting in lower spiritual well‐being. This study revealed how adverse early life experiences can affect spiritual well‐being through an individual's internal resources and cognitive processes. These results suggest that decreasing rumination and cultivating forgiveness are important to alleviate the influence of adverse childhood experiences on spiritual well‐being.

## Author Contributions

**Gülçin Güler Öztekin**: conceptualization, methodology, software, formal analysis, data curation, visualization, writing – original draft. **Hurizat Hande Turp**: conceptualization, investigation, writing – original draft, project administration. **Najmah Abdallah Alzahrani**: writing – review and editing. **Juan Gómez‐salgado**: writing – review and editing. **Murat Yildirim**: writing – original draft, writing – review and editing, supervision, resources.

## Conflicts of Interest

The authors declare no conflicts of interest.

## Ethics Statement

All procedures performed in studies involving human participants were in accordance with the ethical standards of the institutional and/or national research committee and with the 1964 Helsinki Declaration and its later amendments or comparable ethical standards. The ethical approval was obtained from the ethical review board of Agri Ibrahim Cecen University (reference number: E‐95531838‐050.99‐124705). We confirm that all participants provided informed consent before taking part in the study. This ensures that they were fully aware of the study's purpose, procedures, potential risks, and their right to withdraw at any time.

## Peer Review

The peer review history for this article is available at https://publons.com/publon/10.1002/brb3.70923

## Data Availability

The datasets generated and/or analyzed during the current study are not publicly available but are available from the corresponding author upon reasonable request.
